# Knockout of high-mobility group box 1 in B16F10 melanoma cells induced host immunity-mediated suppression of in vivo tumor growth

**DOI:** 10.1007/s12032-022-01659-2

**Published:** 2022-02-12

**Authors:** Kanako Yokomizo, Kayoko Waki, Miyako Ozawa, Keiko Yamamoto, Sachiko Ogasawara, Hirohisa Yano, Akira Yamada

**Affiliations:** 1 Cancer Vaccine Development Division, Research Center for Innovative Cancer Therapy, Kurume University, Kurume, Fukuoka 830-0011 Japan; 2 Department of Pathology, Kurume University School of Medicine, Kurume, Fukuoka 830-0011 Japan

**Keywords:** HMGB1, DAMPs, Tumor microenvironment, High-mobility group box 1, Anti-tumor immunity

## Abstract

High-mobility group box 1 (HMGB1) has been reported as a damage-associated molecular pattern (DAMP) molecule that is released from damaged or dead cells and induces inflammation and subsequent innate immunity. However, the role of HMGB1 in the anti-tumor immunity is unclear since inflammation in the tumor microenvironment also contributes to tumor promotion and progression. In the present study, we established HMGB1-knockout clones from B16F10 and CT26 murine tumors by genome editing using the CRISPR/Cas9 system and investigated the role of HMGB1 in anti-tumor immunity. We found that (1) knockout of HMGB1 in the tumor cells suppressed in vivo, but not in vitro, tumor growth, (2) the suppression of the in vivo tumor growth was mediated by CD8 T cells, and (3) infiltration of CD8 T cells, macrophages and dendritic cells into the tumor tissues was accelerated in HMGB1-knockout tumors. These results demonstrated that knockout of HMGB1 in tumor cells converted tumors from poor infiltration of immune cells called “cold” to “immune-inflamed” or “hot” and inhibited in vivo tumor growth mediated by cytotoxic T lymphocytes. Infiltration of immune cells to the tumor microenvironment is an important step in the series known as the cancer immunity cycle. Thus, manipulation of tumor-derived HMGB1 might be applicable to improve the clinical outcomes of cancer immunotherapies, including immune checkpoint blockades and cancer vaccine therapies.

**Supplementary Information:**

The online version contains supplementary material available at 10.1007/s12032-022-01659-2.

## Introduction

High mobility group box 1 (HMGB1) is one of the major chromatin-associated non-histone proteins in the nucleus and acts as a DNA chaperone [1]. The tissue distribution of HMGB1 is ubiquitous, and malignant cells are also known to express HMGB1 [1, 2]. In addition to its roles in the nucleus and the cytosol, HMGB1 has been reported to be a damage-associated molecular pattern (DAMP) molecule that is released from damaged or dead cells and induces inflammation and subsequent innate immunity [1]. Toll-like receptor (TLR)-2 and -4, the receptor for advanced glycation end products (RAGE), and T cell immunoglobulin and mucin-domain containing-3 (TIM-3) have been identified as cell surface receptors for HMGB1 [1]. Binding of HMGB1 to TLR-2, TLR-4, or RAGE on macrophages and dendritic cells (DCs) induces production of proinflammatory cytokines through NF*k*B activation and/or type 1 interferon production through activation of the transcription factor interferon regulatory factor-3 (IRF3) [1, 3]. These cytokines activate innate immunity and positively contribute to the induction of subsequent adaptive immunity [3]. Thus, HMGB1 would seem to play a positive role in host defense; on the other hand, it is known that inflammation, including HMGB1-induced inflammation, in the tumor microenvironment contributes to tumor promotion and progression [4]. In addition, HMGB1 binding to TIM-3 on tumor-infiltrating DCs negatively contributes to the induction of innate immunity—i.e., TIM-3-mediated signals inhibit nucleic acid-sensing TLRs, such as TLR-9, which mediates the production of proinflammatory cytokines, and thus binding of HMGB1 to TIM-3 has a negative effect on proinflammatory cytokine production [5]. Therefore, the role of HMGB1 in anti-tumor immunity is complicated, and it is unclear whether HMGB1 has a favorable or unfavorable impact on the host defense against tumors [4].

To clarify this issue, we established HMGB1-knockout clones from B16F10 and CT26 tumor cells by genome editing using the clustered regularly interspaced short palindromic repeats (CRISPR)/Cas9 system, and used these clones to investigate the role of HMGB1 on the anti-tumor immunity.

## Materials and methods

### Mice

Seven-week-old female C57BL/6J (B6), BALB/c, and BALB/c-nu/nu mice were purchased from CLEA Japan (Tokyo, Japan) and housed under specific pathogen-free conditions at 22 ± 2 °C, 60 ± 10% humidity, with 12 h light/dark cycle in the animal facility of the Kurume University School of Medicine and provided food pellets and water ad libitum. All animal experimental protocols were approved by the Institutional Animal Care and Use Committee of Kurume University (approval no. 2020-020) in accordance with the national guidelines on the care and use of laboratory animals. In tumor transplantation experiments, tumor size was measured every 2 or 3 days. Experimental groups and the number of mice in each group were as follows: Gene expression analyses consisted of wild type (WT) and two HMGB1-knockout (KO) clones (*n* = 3); tumor growth analyses consisted of WT and three HMGB1-KO clones in B6 mice (*n* = 5); tumor growth analyses consisted of WT and two HMGB1-KO clones in BALB/c mice (*n* = 7); tumor growth analyses consisted of WT and two HMGB1-KO clones in nu/nu mice (*n* = 5); tumor growth analyses consisted of WT and two HMGB1-KO clones in T cell subset-depleted mice, total 12 groups (*n* = 5); tumor growth analyses consisted of WT, one HMGB1-KO clone and their HMGB1 transfectants in B6 mice (*n* = 7); immunohistochemistry consisted of WT and two HMGB1-KO clones (*n* = 6); tumor growth analyses consisted of WT, two HMGB1-KO clones, mixture of WT and HMGB1-KO clones or co-transplanted without mix, total 9 groups in B6 mice (*n* = 7); tumor growth analyses consisted of WT and one HMGB1-KO clone made by AAV in the supplement data (*n* = 7). Humane endpoints in this study were as follows: (1) tumor size reached > 20 mm in a diameter, (2) lethargic condition. Otherwise, mice were sacrificed via cervical dislocation at the indicated periods or at 60 days after tumor transplantation to obtain tumor and lymphoid tissue.

### Establishment of HMGB1-knockout cells

Murine melanoma B16F10 cells were purchased from ATCC through Sumitt Pharmaceuticals (Tokyo, Japan). Colon tumor Colon-26 (CT26) cells were originally obtained from Dr. K. Nomoto, Kyushu University (Fukuoka, Japan) and maintained in our laboratory. These cells were cultured in D-MEM (high glucose) (Fujifilm, Tokyo, Japan) and RPMI 1640 (Nacalai Tesque, Kyoto, Japan) supplemented with 10% FCS (Thermo Fisher), l-glutamine and 50 μg/ml gentamicin at 37 °C in a 5% CO_2_ incubator. Knockout of the HMGB1 gene in B16F10 and CT26 cells was performed by using a Hmgb1-KN2.0 mouse gene knockout CRISPR kit (KN507817; Origene, Rockville, MD) according to the manufacturer’s instruction. B16F10 and CT26 cells were transfected with pCas-Guide CRISPR vector containing HMGB1 guide RNA (gRNA) and linear donor EF1a-GFP-P2A-Puro using Xfect Transfection Reagent (Takara Bio, Kusatsu, Japan) or a K2 Transfection System (Biontex, Munchen, Germany). The target sequences of the gRNA vectors were as follows: Vector 1, 5′-GGAGATCCTAAAAAGCCGAG-3′ and vector 2, 5′-CTCCCCTTTGGGGGGGAYGT-3′, targeting the exon 2 and 3, respectively. The linear donor contains stop codon, thus the insertion of the linear donor at the editing site in the exons 2 and 3 disrupts all the functional domains of HMGB1. Puromycin selections (1 µg/ml) of the cells were performed at 10, 14 or 21 days after the transfection. Single cell colonies were obtained by limiting dilution after the puromycin selection. Knockout of the HMGB1 gene was confirmed by Western blot analysis. The knockout clones were maintained under the presence of puromycin (1 µg/ml) and used for further experiment before one month of in vitro culture. All gene modification experimental protocols were approved by the Institutional Genetic Modification Safety Committee of Kurume University (approval no. 30-11) in accordance with the national guidelines for research involving recombinant DNA experiments.

### Transduction of the HMGB1 gene

The mouse HMGB1 gene was stably transduced to the knockout cells or wild-type cells using a Lentivirus system. Preparation of viral vector was as follows: 1 μg of a mouse HMGB1 lentiviral cDNA ORF clone, pLV-mHMGB1-GFPSpark (Sino Biological) and 1 μg 3rd Generation Packaging Mix (Applied Biological Materials) was transfected into 5 × 10^5^ 293 T cells (Takara Bio) using 6 μl TransIT-Lenti transfection reagent (Mirus Bio, LLC) at 37 °C for 48 h according to the manufacturer’s instruction. Recombinant virus was concentrated by Lenti-X Concentrator (Takara Bio), checked for its titer using the Lenti-X GoStix Plus (Takara Bio) and stored at − 80 °C until use. Lentiviral vector (3800 GoStix values compatible to 9.8 × 10^4^ infection units) was transduced into 4 × 10^4^ cells (MOI = 2.5) of knockout clones or WT cells with 8 μg/ml polybrene at 37 °C for 48 h. Then, the culture medium was changed to fresh medium not containing polybrene and the cells were diluted and put into a well of 96-well plate (0.2 cells/well) for single cell cloning. Expression of HMGB1-GFP fusion protein was confirmed by western blot analysis. After confirmation of HMGB1 expression, the cells were expanded and used for further study within two weeks of in vitro culture to avoid loss of HMGB1 expression.

### Western blotting

Cells were lysed by RIPA buffer (Thermo Fisher) with 1 × protease inhibitor cocktail (Nacalai Tesque) for 5 min on ice, then sonicated and centrifuged at 14,000×*g* for 15 min at 4 °C to remove the cell pellet. The protein concentration of the lysate was measured by using a BCA protein assay kit (Thermo Fisher), and 8 μg of protein for each lysate was mixed with 4 × NuPAGE LDS Sample Buffer (Thermo Fisher) and 10 × NuPAGE Reducing Agent containing 0.5 M dithiothreitol, denatured at 70 °C for 10 min, and subjected to 12% SDS-PAGE. After electrophoresis, the proteins were transferred to an Immobilon-P membrane (Merk Millipore, Darmstadt, Germany). The membrane was blocked with Blocking One (Nacalai Tesque) at 4 °C overnight and then incubated with 1:4000 rabbit anti-HMGB1 antibody (cat. no. ab18256) (Abcam, Cambridge, UK). After washing with 0.1% Tween 20-Tris-buffered saline (TBST), the membrane was incubated with 1:5000 horseradish peroxidase-conjugated anti-rabbit IgG (Abcam; cat. no. ab6721) for 1 h at room temperature (r.t.) and rinsed with TBST 3 times. Detection was performed using Clarity Western ECL Substrate (Bio-Rad, Hercules, CA) and LAS-4000 mini (Fujifilm). The band intensities were analyzed using MultiGauge ver 3.0 (Fujifilm). The expression of HMGB1 protein was normalized to the amount of loading protein or α-tubulin using anti-α-tubulin pAb-HRP DirecT (1:3000; MBL, cat. no. PM054-7). Rabbit anti-GFP antibody (1:2000; Abcam; cat. no. ab290) was used to detect the HMGB1-GFP fusion protein.

### Assessment of in vitro cell proliferation and glucose consumption

HMGB1-knockout or wild type (WT) cells were placed into the wells of a 96-well plate (3500, 1750, and 875 cells/well), and the live cell number in the culture was counted daily by using a cell counting kit-8 (Dojindo, Kumamoto, Japan). To determine glucose consumption, 1 × 10^4^ of HMGB1-knockout or WT cells were placed into a well of a 96-well plate. The glucose concentration of the culture supernatant of cells was determined by a Glucose Assay Kit-WST (Dojindo) and the glucose consumption was calculated using the following formula:

$${\text{Glucose}}\,{\text{consumption}}\, = \,\left( {{\text{glucose}}\,{\text{concentration}}\,{\text{of}}\,{\text{fresh}}\,{\text{medium}}} \right)\, - \,\left( {{\text{glucose}}\,{\text{concentration}}\,{\text{of}}\,{\text{ 1 - or 2 - day}}\,{\text{culture}}\,{\text{supernatant}}} \right)$$

Data from triplicate assays were plotted for each study. Two independent experiments were done.

### Assessment of in vivo tumor growth

1 × 10^6^ of WT or HMGB1-knockout cells, or the mixtures containing WT and HMGB1-knockout cells at ratios of 1:1 and 1:3 were subcutaneously (s.c.) injected to the flanks of mice, and tumor size was measured every 2 or 3 days using a caliper. Tumor volumes were calculated using the following formula:

$${\text{Tumor}}\,{\text{volume}}\,\left( {{\text{mm}}^{{3}} } \right)\, = \,\left( {{\text{Greatest}}\,{\text{longitudinal}}\,{\text{diameter}}} \right) \times \left( {\text{greatest transverse diameter}} \right)^{{2}} \, \times \,0.{5}$$

Two independent experiments using 5–7 mice per group were performed.

### In vivo depletion of T-cell subsets

Mice were intraperitoneally injected total three times with 0.25 mg/mouse of anti-CD4 (clone GK1.5), anti-CD8 (clone 53.6.72) or anti-CD25 (clone PC-61.5.3) monoclonal antibodies (mAbs), all from Bio X Cell (Lebanon, NH), on the day of tumor inoculation (day 0) and days 4 and 8. Depletions of the corresponding cell populations using this protocol were confirmed by a flowcytometric analysis of spleen cells obtained one day after the mAb inoculation (data not shown). Two independent experiments with 5 mice per group were performed.

### Gene expression analysis

Multi-gene expression in the tumor tissues was analyzed by nCounter digital analyzer (nCounter SPRINT) with PanCancer Mouse Immune Profiling panel (both NanoString Technologies, Inc.) containing 750 cancer-associated immunity-related mouse genes. Freshly obtained tumor specimens were soaked in RNAlater (Thermo Fisher) and minced, and the mRNA was further purified using an RNeasy Plus Universal Kit (QIAGEN, Venlo, Netherlands) according to the manufacturer’s instruction. The obtained mRNA samples were subjected to nCounter analysis and then further analyzed using nSolver v4.0 (NanoString) software.

### Immunohistochemistry

Tissue specimens were fixed in 10% neutral buffered formalin for ~ 18 h, paraffin-embedded, and cut into 4 μm sections. The sections were subsequently transferred to glass slides, deparaffinized, and rehydrated. Antigen retrieval was performed in 1 mM EDTA-10 mM Tris–HCl (pH9.0) at 110 °C for 30 min using a decloaking chamber (NxGen; Biocare Medical, Pacheco, CA). After blocking of endogenous peroxidase with BLOXALL (Vector Laboratories, Burlingame, CA) followed by 2.5% normal horse or goat serum for 20 min, the sections were further incubated with the first antibodies at r.t. for 1 h or at 4 °C overnight, rinsed twice with 0.1% Tween 20-phosphate-buffered saline for 5 min, and incubated with the second antibodies at r.t. for 30 min. Chromogenic detection was performed using HistoGreen (HISTOPRIME, Linaris Biologische Produkte, Mannheim, Germany). Slides were counterstained with Vector Hematoxylin QS (Vector Laboratories). The antibodies used for the immunostaining were as follows: rabbit mAbs against mouse CD4 (1:500; cat. no.EPR19514; Abcam), CD8α (1:500; cat. no. EPR20305; Abcam), F4/80 (1:350; cat. no. D2S9R; Cell Signaling Technology, Tokyo, Japan), and CD11c (1:200; cat. no. D1V9Y; Cell Signaling Technology), and rat mAb against mouse Foxp3 (1:50; cat. no. FJK-16s; eBioscience). Peroxidase-conjugated horse anti-rabbit IgG and anti-rat IgG polymer kits (ImmPRESS; Vector Laboratories; cat. no. MP-7801 and MP-7444) were used as the second antibodies. For the staining of CD11c, anti-CD11c mAb was incubated at 4 °C overnight, and Boost IHC detection reagent (HRP, rabbit; Cell Signaling Technology) was used as the second antibody for 30 min at r.t., following the 15 min incubation at r.t. with EnVision FLEX + rabbit Linker (Agilent, Santa Clara, CA).

### Euthanasia

To obtain tumor tissues or lymphoid tissues, the mice were euthanized by cervical dislocation.

### Statistical analysis

**Fig. 1:**
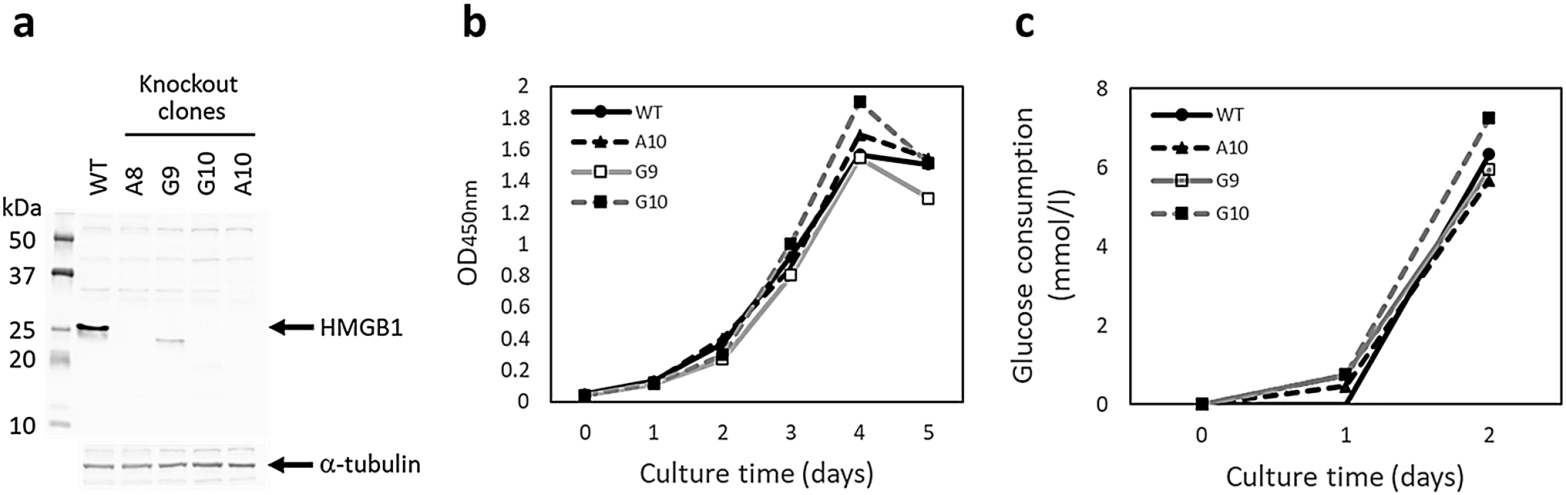
Establishment of HMGB1-knockout clones of B16F10 cells. **a** Western blot analysis of HMGB1-knockout clones of B16F10 cells. A 25 kDa band of HMGB1 protein found in the wild type B16F10 cells is not detected in knockout clones. α-tubulin was used as an internal control. Experiments for each clone were repeated at least twice during screening and representative results are shown. **b** In vitro cell proliferation of HMGB1-knockout clones of B16F10 cells. Experiments were performed in triplicate and representative results (875 cells/well) of the three independent experiments are shown. *WT* wild type cells. There was no statistically significant difference in cell growth between the WT and each HMGB1-knockout clone. **c** Glucose consumption of HMGB1-knockout clones of B16F10 cells. Experiments were performed in triplicate and representative results of the two independent experiments are shown. Glucose consumption was not significantly different between the WT and each HMGB1-knockout clone

**Fig. 2:**
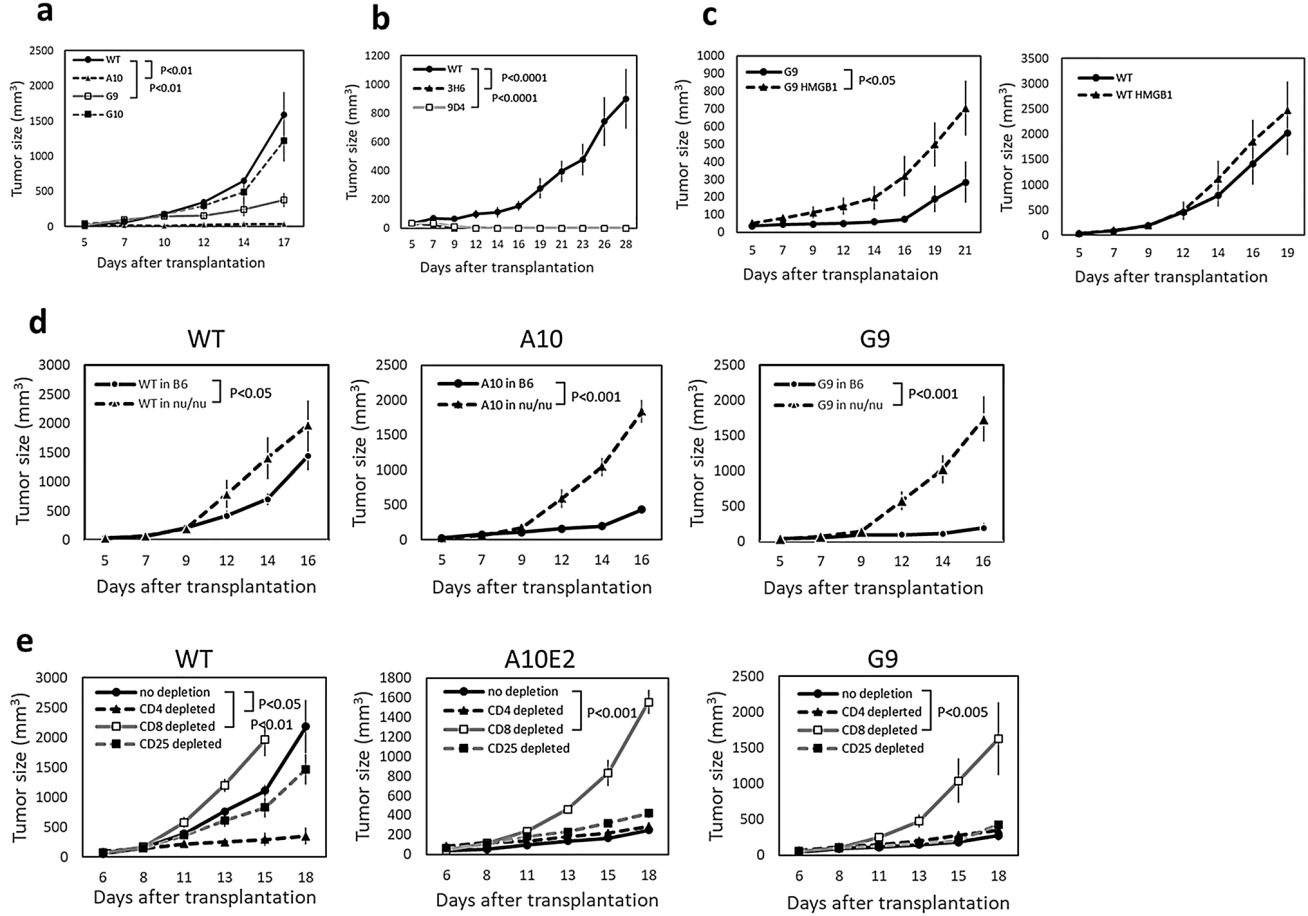
In vivo tumor growth of HMGB1-knockout clones. Representative results of at least two experiments are shown. **a** Tumor growth of wild type (WT) and HMGB1-knockout clones (A10, G9, G10) of B16F10 cells after s.c. transplantation to B6 mice is shown (*n* = 5 per group). **b** Tumor growth of wild type (WT) and HMGB1-knockout clones (3H6, 9D4) of CT26 cells after s.c. transplantation to BALB/c mice is shown (*n* = 7). **c** The HMGB1 gene was stably transduced to G9 cells or WT B16F10 cells and in vivo tumor growth was examined in B6 mice (*n* = 7). **d** Tumor growth of WT B16F10 cells and knockout clones (A10, G9) in athymic nu/nu and control B6 mice is shown (*n* = 5). **e** Tumor growth of WT B16F10 cells and knockout clones (A10E2, G9) in CD4, CD8, or CD25 cell-depleted mice or control B6 mice (no depletion) is shown (*n* = 5). The error bars represent the standard error of the mean

**Fig. 3:**
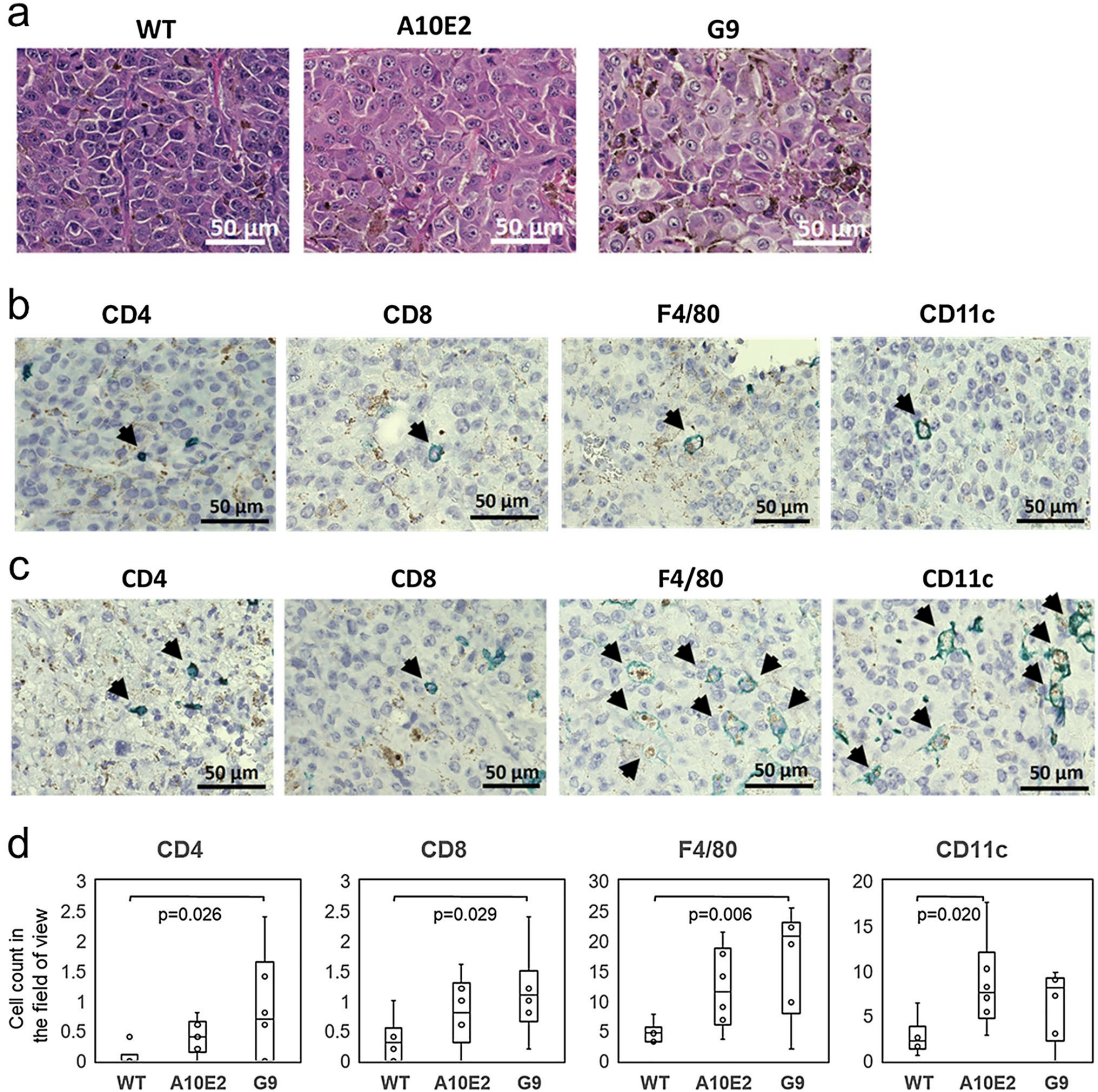
Representative images of tumor tissues 14 days after transplantation. Original magnification of the objective lens was × 20. **a** Hematoxylin–eosin staining of WT, A10E2, and G9 tumor tissues. **b, c** Representative images of the immunohistochemistry of WT (**b**) and A10E2 (**c**) tumor tissues. Arrows indicate positively stained cells. **d** Counts of CD4, CD8, F4/80, or CD11c positive cells in the field of view of the WT, A10E2, and G9 tumor tissues (*n* = 6 per group). The error bars represent the standard error of the mean

**Fig. 4:**
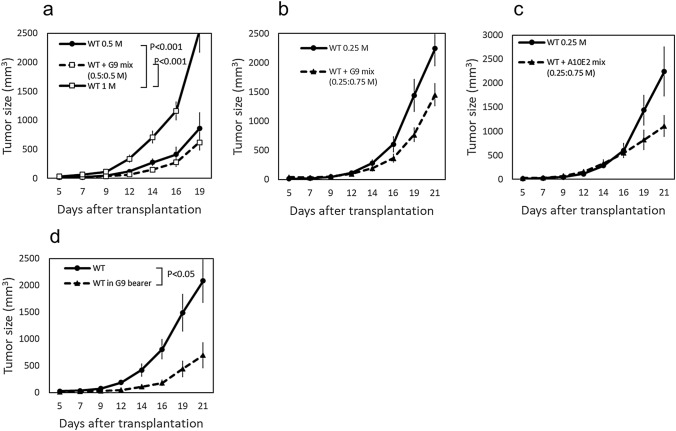
Effect of co-transplanted HMGB1-knockout tumor on in vivo growth of WT B16F10 cells. Representative results of at least two experiments are shown. Each group included 7 mice. **a** 0.5 × 10^6^ WT cells alone or mixed with 0.5 × 10^6^ G9 cells were s.c. transplanted to B6 mice. The tumor growth of 1 × 10^6^ WT cells transplanted alone is also shown. **b** 0.25 × 10^6^ WT cells alone or mixed with 0.75 × 10^6^ G9 cells were s.c. transplanted to B6 mice. **c** 0.25 × 10^6^ WT cells alone or mixed with 0.75 × 10^6^ A10E2 cells were s.c. transplanted to B6 mice. **d** 0.25 × 10^6^ WT cells were s.c. transplanted to the right flank of B6 mice, and 0.75 × 10^6^ G9 cells were simultaneously transplanted to the left flank of the same mice (G9 bearer). The tumor growth of WT cells in the G9 bearer or in intact B6 mice is shown. The error bars represent the standard error of the mean

Differences between two groups were analyzed as follows: In Fig. 1b, c and 3d, the data were analyzed by one-way ANOVA followed by Tukey’s post hoc test. In Figs. 2a, 4 and S1, the data were analyzed by two-way mixed ANOVA (full factorial repeated measures ANOVA) followed by Tukey’s post hoc test (Figs. 2a, b, e, and 4a) or t-test (Figs. 2c, d, 4b–d, and S1). A *p* value < 0.05 was considered statistically significant. Statistical analyses were performed using JMP Pro version 15 software (SAS Institute, Cary, NC). In the immunohistochemistry, positive cell counts in an average of 5 continuous fields of view of the tissue specimens from 6 mice per group were compared with each other.

## Results

### Establishment and characteristics of HMGB1-knockout clones

B16F10 cells were transfected with pCas-Guide CRISPR vector containing HMGB1 gRNA and linear donor EF1a-GFP-P2A-Puro for the genome editing. We used two gRNA vectors, and only one gRNA vector (gRNA vector 1) worked well in the B16F10 cells (data not shown). After single cell cloning, four knockout clones were obtained. Western blot analysis confirmed that the HMGB1 gene in the four clones (A8, A10, G9, G10) was completely knocked out (Fig. 1a). The in vitro cell proliferation of the HMGB1-knockout clones (A10, G9, G10) was further compared with that of the wild type (WT) B16F10 cells; however, there were no significant differences between each clone and the WT cells (Fig. 1b). It is known that HMGB1 regulates transcription of numerous genes as a DNA-binding protein [1] and may affect subsequent expression of proteins and metabolism. Therefore, we also analyzed glucose metabolism of the cells, and again there were no significant differences between each knockout clone and the WT cells (Fig. 1c).

### In vivo tumor growth of the HMGB1-knockout clones

The knockout clones and WT B16F10 cells were s.c. transplanted to B6 mice and the subsequent tumor growth was analyzed. As shown in Fig. 2a, the tumor growth of the two clones (A10, G9) in B6 mice was significantly slower than that of WT B16F10 cells. The tumor growth of clone G10 was slightly slower than that of WT cells but with no statistical significance. The color of the cell pellet, which reflected melanin content, of clone G10 appeared to be different from those of the other clones (data not shown), suggesting that the clonal heterogeneity of the parental B16F10 cells might have influenced the results. To determine whether the in vivo growth inhibition found in HMGB1-knockout clones was a general phenomenon caused by HMGB1 knockout, we established two HMGB1-knockout clones (3H6, 9D4) from CT26 colon tumor cells and compared their in vivo tumor growth in BALB/c mice with that of WT CT26 cells (Fig. 2b). The tumor growth of both the 3H6 and 9D4 clones was markedly suppressed and seemed to have disappeared in most mice. Similar in vivo tumor growth inhibition was also observed in the HMGB1-kockout clones established from B16F10 cells using an adeno-associated virus vector and an *S. aureus* Cas9 system (AAVpro CRISPR/SaCas9; Takara) with different gRNA (Fig. S1). These results suggested that the in vivo tumor growth inhibition was a general phenomenon caused by HMGB1 knockout. Re-expression of HMGB1 by stable transfection of the HMGB1 gene in the knockout clones enhanced the in vivo tumor growth (Fig. 2c). For further analyses, we used clones G9 and A10 as representatives of HMGB1-knockout clones derived from B16F10 cells. In some experiments, we also used the A10-derived subclone A10E2, which represented the dominant population of A10, since A10 contained a minor population with different autofluorescence (data not shown).

The contribution of host immunity to the discrepancy between the in vitro and in vivo tumor growth of HMGB1-knockout clones was further analyzed using athymic nu/nu mice. The tumor growth suppression of A10 and G9 found in B6 mice was not observed in nu/nu mice (Fig. 2d). These results suggested that host T-cell-mediated immunity contributed to in vivo tumor growth inhibition of knockout clones. To clarify the contribution of host immunity, in vivo depletion of the CD4, CD8, or CD25 T-cell subset was performed (Fig. 2e). In the CD4-depleted mice, tumor growth of the WT cells was suppressed. In contrast, in the CD8-depleted mice, tumor growth of the WT cells was augmented. Similar tumor growth augmentation by CD8-depletion was also found in the mice transplanted with A10E2 and G9 cells. These results indicated that the in vivo tumor cell growth inhibition found in HMGB1-knockout cells was mediated by CD8 T-cells.

### Expression of immune-related genes in the tumor tissues

Comprehensive expression analysis of 750 immune-related genes in the tumor tissues of B6 mice at 14 days after transplantation of A10E2, G9, and WT B16F10 cells was performed using an nCounter PanCancer mouse immune profiling panel. The expressions of 28 and 130 genes in the 750 gene panels in the G9 tumor tissue were increased more than 10- and fivefold over the levels in WT B16F10 tumor tissue, respectively. Table 1 shows the expression of the top 50 overexpressed genes found in the G9 tissue along with the corresponding expression levels in the A10E2 tumor tissues. All 50 of the most overexpressed genes in the G9 tissue were also highly expressed in the A10E2 tumor tissues, and thus the high expression of the 50 genes in the tumor tissues was a common phenomenon in HMGB1-knockout tumors. The expression of 28 of the 50 genes suggested the presence of tumor-infiltrated macrophages in the HMGB1-knockout tumors, while the expression of 12 of the 50 genes suggested infiltration of T cells into the tumor tissues. In addition, the top 50 overexpressed genes contained 10 chemokine-ligands. These data suggested that the knockout of HMGB1 induced infiltration of macrophages and T cells into the tumor tissues.

**Table 1:** Top 50 overexpressed genes found in the HMGB1 knockout tumor tissues

Protein/gene	Expression index (Clone/WT)		Annotations			Functions
			Expected expressing cells			
	G9	A10E2	Macrophages	T cells (activated)	Others	Chemokine/ligand
CCL8	48.7	13.2	+ (M1)			+
C3	34.2	9.4	+			
LYZ2	18.1	4.7	+			
CTSS	16.6	3.8	+		DC	
CXCL12	16.2	14.8			Ubiquitous	+
CCL5	14.5	2.7	+ (M1)			+
MARCO	13.5	6.4	+			
CCL11	13.4	4.7	+ (M1)			+
CD84	13.2	4.3	+		B	
H2-I-E	12.9	6.4	+	+	B	
CfB	12.8	7.0			Ubiquitous	
CD74	12.5	5.9	+		B	
PD-L2	12.4	2.5	+			
MRC1/CD206	12.3	5.0	+ (M2)			
Cybb	12.2	3.0	+		PMN	
IL2Rb	12.1	4.0		+		
CD45	11.9	6.2	+	+	Leukocytes	
COL3A1	11.9	8.8			Connective tissues	
CD3g	11.8	5.2		+		
H2-I-Aa	11.7	5.4	+	+		
H2-I-Ab1	11.5	5.1	+	+		
KLRD1/CD94	11.5	4.1			NK	
CD180	11.5	3.9			B	
SLAMF7/CD319	11.4	4.1			Plasma cells	
TNFR2	11.0	4.8	+			
Chil3	10.8	1.3	+ (M2)		+	
CD48	10.6	4.7			B	
ABCG1	10.0	5.5	+			
Cfh	9.8	6.9			Ubiquitous	
Xcl1	9.7	1.9		CD8		+
Emr1/ F4/80	9.5	4.5	+			
Serping1	9.5	9.4	+		DC, neutrophils	
Gzma	9.4	1.3		CD8	NK	
Ly9/CD229	9.3	2.4		+	Lymphocytes	
Cxcr6	9.3	2.5		+	Ubiquitous	+
Selplg	9.2	3.2		+	Myeloid cells	
Lbp/CD14	8.8	10.4	+		Macrophage	+
Csf3r	8.7	4.6			Neutrophils	
Col1a1	8.7	9.3			Ubiquitous	
Klrc1	8.7	2.0			NK	
Klrk1	8.6	2.2			NK	
C3ar1	8.6	4.8			Ubiquitous	
Csf1r	8.3	4.2	+			+
F13a1	8.3	8.5	+			
Ccl6	8.1	2.7	+		Neutrophils	+
Cd200r1	8.0	2.8	+ (M2)		B	
Sell	8.0	2.5			Leukocytes	
Abca1	7.9	3.0	+			
Cfd	7.9	8.9			Ubiquitous	
Ccr5	7.8	4.2	+	+		+
Total	50	50	28	12	28	10

*B* B cells; *DC* dendritic cell; *M1* type 1 macrophages; *M2* type 2 macrophages; *NK* natural killer cells; *PMN* polymorph nuclear cells

Next, we focused on the gene expression of the anti-tumor immunity-related genes (Table 2). The expression levels of immune suppression-associated genes such as Foxp3, indoleamine-2,3-dioxygenase (IDO1), interleukin-10 (IL-10), and transforming growth factor-β (TGF-β) in the HMGB1-knockout tumor tissues were almost equivalent or slightly increased compared to the corresponding levels in the WT tumor tissues. The single exception was arginase 1 (Arg-1), a marker of myeloid-derived suppressor cells (MDSCs), which was expressed more highly in the HMGB1-knockout tumor tissues than the WT tumor tissues. The expression levels of the genes positively associated with anti-tumor immunity, such as CD8, interferon-γ (IFN-γ), and perforin, in the HMGB1-knockout tumor tissues were higher than those in the WT tumor tissues. These results suggested that the infiltration of immunosuppressive cells, such as regulatory T cells (Treg) and MDSCs, into the tumor microenvironment was not different between the HMGB1-knockout tumor and WT tumor tissues. In contrast, the infiltration of anti-tumor effector cells, such as cytotoxic T-lymphocytes (CTLs) and natural killer (NK) cells, into the tumor microenvironment was accelerated in the HMGB1-knockout tumors.

**Table 2:** Expression of anti-tumor immunity-related genes

Protein/gene	Expression index (Clone/WT)		Expected expressing cells
	G9	A10E2	
Negatively associated			
Arg-1	5.7	2.2	MDSC
TGF-β1	2.3	1.7	Treg, MDSC
IDO1	1.4	0.9	MDSC
Foxp3	1.3	1.2	Treg
IL-10	1.2	0.8	Treg
Positively associated			
Perforin 1	5.9	2.7	CTL, NK
CD8α	5.5	1.5	CTL, NK
IFN-γ	3.1	1.7	CTL
CD8β	3.1	1.2	CTL

### Immunohistochemistry of tumor tissues

The tissues of tumors formed by transplantation of the WT B16F10, A10E2 and G9 cells at 14 days after s.c. transplantation were subjected to an immunohistochemical analysis. Hematoxylin–eosin staining showed marked morphology changes in the A10E2 and G9 tumor tissues, i.e., the majority of tumor cells changed to large cells with a low nuclear-cytoplasmic ratio (Fig. 3a). These morphology changes were not observed when the cells were cultured in vitro (data not shown). Next, the tissue specimens were stained with CD4, CD8, F4/80, CD11c, or Foxp3 mAbs. Representative images of the immunohistochemistry of the WT B16F10 tumor tissues are shown in Fig. 3b. Small numbers of CD4^+^ or CD8^+^ T cells were found in the WT tissues. Infiltration of these T-cell subsets into the tumor tissues was increased in the A10E2 and G9 tumor tissues (Fig. 3c). The average number of the CD4 and CD8 cells in the fields of view of the WT, A10E2 and G9 tissues are shown in Fig. 3d. In contrast to the CD4 and CD8 T cells, F4/80^+^ macrophages and CD11c^+^ DCs were more abundant in the WT tissue and increased in the A10E2 and G9 tumor tissues. Foxp3^+^ cells, containing majority of Treg, were occasionally found in the WT tissue or in the A10E2 and G9 tissues (data not shown).

### Effect of co-transplanted HMGB1-knockout tumor on the in vivo growth of wild-type tumor

To determine whether the in vivo growth of the tumor made up of WT cells was suppressed when the cells were mixed with HMGB1-knockout clones, a total of 1 × 10^6^ cells consisting of a mixture of WT and G9 cells in two different ratios, 1:1 and 1:3, were s.c. transplanted to B6 mice (Fig. 4). The tumor growth of the mixtures (total 1 × 10^6^ cells) appeared to be suppressed when compared to that by transplantation of 1 × 10^6^ WT cells. When the tumor growth of the 1:1 mixture (0.5 × 10^6^ of each type of cells) was compared to that of 0.5 × 10^6^ WT cells, the tumor growth of the mixture was slightly slower than that of WT cells (Fig. 4a). Inhibition of the tumor growth seemed more apparent, but not statistically significant, in the 1:3 mixture—i.e., the tumor growth resulting from the transplantation of 0.25 × 10^6^ WT cells plus 0.75 × 10^6^ G9 cells seemed slower than that by transplantation of 0.25 × 10^6^ WT cells alone (Fig. 4b). A similar tendency was observed when the WT cells were mixed with A10E2 cells (Fig. 4c). Next, the WT and G9 cells were simultaneously but separately transplanted into different sites of the same mouse and the tumor growth of the WT cells was examined (Fig. 4d). The tumor growth of the WT cells in the G9 tumor bearers was markedly suppressed when compared with that in the intact mice.

## Discussion

Immunogenic cell death of tumor cells initiates a series of steps known as the cancer immunity cycle [6]. However, in most patients the cancer immunity cycle is not sufficiently robust to prevent the progression of cancer [6]. One of the factors contributing to the inefficiency of the cancer immunity cycle is the release of an insufficient amount of tumor antigens from tumor cells to prime and activate T cells and initiate the cancer immunity cycle [6]. To reinforce this step, our group has focused on the development of peptide-based cancer vaccines over the last two decades. We developed a personalized peptide vaccine, in which 4 peptides are selected and used as vaccines from a candidate peptide panel consisting of 31 CTL-epitope peptides according to the patient’s HLA-A locus type and reactivity against pre-existing immunity [7, 8]. In the early phase of clinical studies, the personalized peptide vaccines exhibited survival benefits in patients with different types of cancer, but because the benefits were limited, we concluded that the vaccines should be used in combination with other treatment modalities to facilitate the cancer immunity cycle.

The immunogenic cell death of tumor cells releases tumor antigens together with various DAMPs into the tumor microenvironment [9]. DAMPs, including HMGB1, are thought to be an initiator of DC maturation and subsequent anti-tumor immunity [1, 2]. In the present study, we found that (1) knockout of HMGB1 in the tumor cells suppressed in vivo, but not in vitro, tumor growth, (2) the suppression of the in vivo tumor growth was mediated by CD8 T cells, and (3) infiltration of CD8 T cells, macrophages, and DCs into the tumor tissues was accelerated in HMGB1-knockout tumors. These results suggest that tumor-derived HMGB1 promotes in vivo tumor growth through the inhibition of anti-tumor immunity by suppressing the infiltration of anti-tumor immune cells into the tumor microenvironment, and therefore, HMGB1 plays a negative role in the host defense against tumors. The results shown in this study were investigated under spontaneous cell death of tumor tissues. If immunogenic tumor cell death were induced by chemotherapy or radiation therapy [10], the effect of HMGB1-knockout could be more apparent.

Recent progress in immune checkpoint blockades (ICBs) has been a leading force in cancer immunotherapy, and immunotherapy is currently a major modality among cancer treatments. However, only a subset of patients exhibit a durable response—i.e., only approximately 10–40% of patients treated with anti- Programmed cell death protein 1 (PD-1)/Programmed death-ligand 1 (PD-L1) antibody monotherapy exhibited a durable response; the remaining majority population received no clinical benefits of ICBs [11–14]. The effectiveness of ICBs is dependent on aspects of the tumor microenvironment, such as the infiltration of immune cells and expression of immune checkpoint molecules [6, 15]. Tumor tissues with enriched infiltration of immune cells, such as CD4 and CD8 T cells, are defined as “immune-inflamed” or “hot” tumors [16, 17]. In contrast, tumor tissues with poor or no infiltration of immune cells are called “non-inflamed” or “cold” tumors. Clinical studies of ICBs indicated that ICBs were more effective in patients with hot tumors than those with cold tumors [15–17]. Our results indicated that knockout of HMGB1 in tumor cells actually converted cold tumors to hot tumors. These results suggest the possibility of applying HMGB1 knockout to ICB therapy. The conversion of cold to hot tumors by HMGB1 knockout may improve the efficacy of ICB therapy.

Several studies have assessed the role of HMGB1 in anti-tumor immunity [18–20]. Liu et al. [18] investigated the effect of HMGB1 knockdown in murine breast cancer 4T1.2-Neu and 3LL lung cancer cells using small interfering RNA. Similar to the present results, their experiments showed that knockdown of HMGB1 in tumor cells did not affect the in vitro tumor growth, but in vivo tumor progression was suppressed by the knockdown and longer overall survival was observed in knockdown tumor animals. They also found that knockdown of HMGB1 attenuated Treg induction and upregulated CD8 T cell-dependent anti-tumor immunity. Zhang et al. [19] reported the involvement of DCs in induction of Treg by tumor-derived HMGB1 and suggested that the underlying mechanism may involve HMGB1 and thymic stromal lymphoprotein, both derived from tumor cells, modulation of DCs to activate Treg and suppress CTL function. They also showed that box A, an antagonist of HMGB1, and glycyrrhizin, a selective inhibitor of HMGB1, inhibited in vivo tumor growth of 4T1.2-Neu cells. Tumor growth inhibition by extracellular HMGB1 blockade through remodeling of immune microenvironment was reported by Hubert et al. [20]. They found reduction of MDSCs and Tregs and increase of M1/M2 ratio and DCs in the tumor microenvironment. They also reported blocking of HMGB1 improved efficacy of anti-PD-1 therapy. Induction of Treg by HMGB1 is also reported in type 1 diabetes [21] and psoriasis vulgaris, an autoimmune inflammatory skin disease [22]. Induction of MDSCs and regulatory B cells by HMGB1 have been reported [23, 24]. Previous studies have mainly focused on the effect of HMGB1 on induction of immunosuppressive cells, such as Treg and MDSCs [19, 20]. By contrast with previous studies, the present study demonstrated the suppressive effect of HMGB1 on infiltration of immune cells into the tumor microenvironment. Promoted infiltration of immune cells in HMGB1-knockout tumor tissues might be a result of mitigation of local immunosuppression by Treg and MDSCs.

In conclusion, we demonstrated that knockout of HMGB1 in tumor cells converted cold tumors to hot tumors and suppressed in vivo tumor growth mediated by CTLs. Infiltration of immune cells to the tumor microenvironment is an important step in the cancer immunity cycle. Thus, the manipulation of tumor-derived HMGB1 might be applicable to improve the clinical outcomes of cancer immunotherapies including ICB and cancer vaccine therapies.

## Supplementary Information

Below is the link to the electronic supplementary material.

Supplementary file1 (TIF 435 KB)
Figure S1. In vivo tumor growth of HMGB1-knockout clone D8D3 established from B16F10 cells using an adeno-associated virus vector and S. aureus Cas9 system (AAVpro CRISPR/SaCas9 vector system; Takara). Tumor growth of wild type (WT) and HMGB1-knockout clone D8D3 of B16F10 cells after s.c. transplantation to B6 mice is shown. Each group included 7 mice. The error bars represent the standard error of the mean. (Supplementary materials)

## Data Availability

The data used to support the findings of this study are available from the corresponding author upon reasonable request.
