# Supplementary figures and images for: Knockout of high-mobility group box 1 in B16F10 melanoma cells induced host immunity-mediated suppression of in vivo tumor growth
Source: Med Oncol. 2022 Feb 12;39(5):58. doi: 10.1007/s12032-022-01659-2 (PMC8840913; doi:10.1007/s12032-022-01659-2)

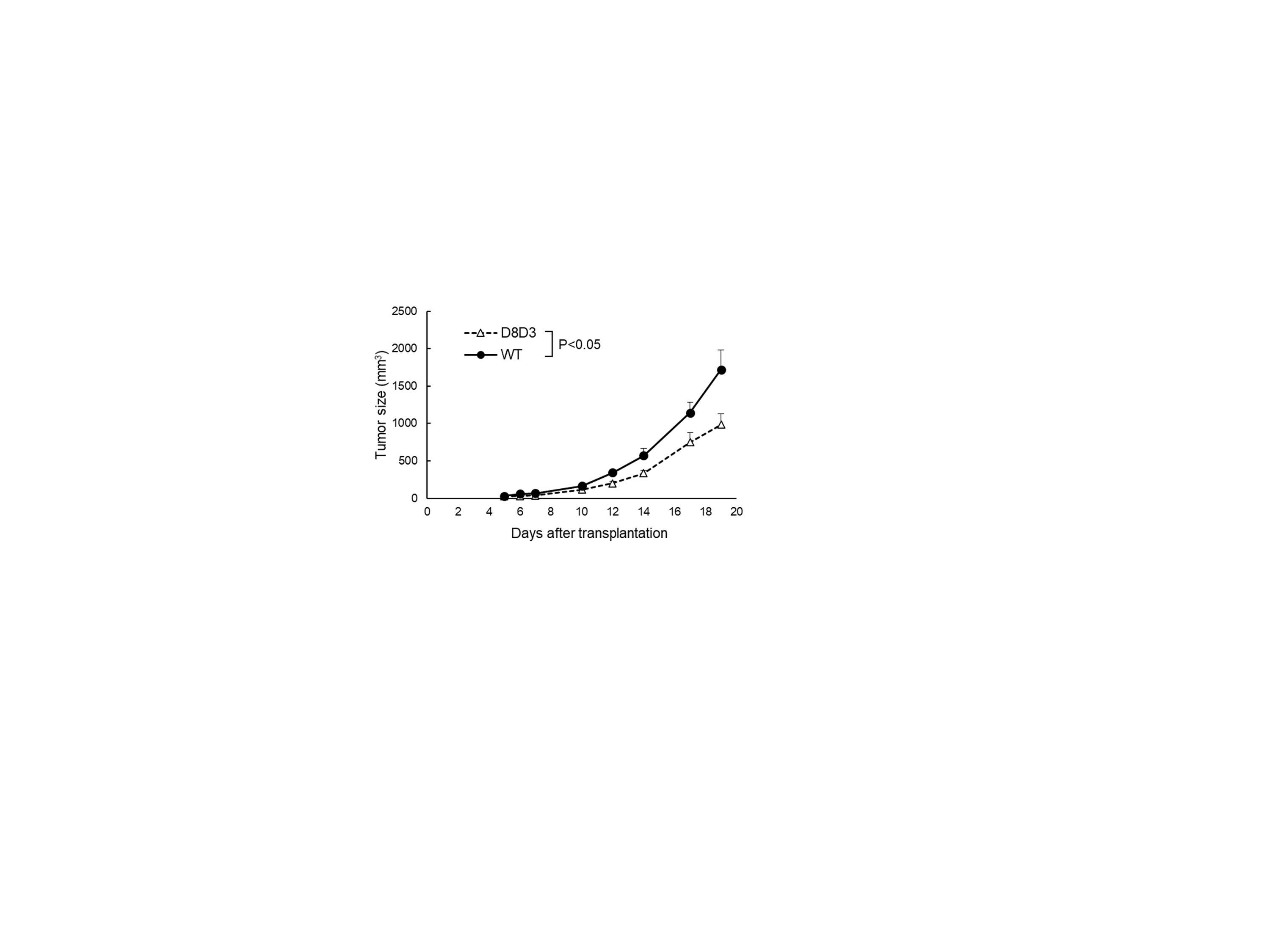

Supplement: Supplementary file 1 — Supplementary file1 (TIF 435 KB) Figure S1. In vivo tumor growth of HMGB1-knockout clone D8D3 established from B16F10 cells using an adeno-associated virus vector and S. aureus Cas9 system (AAVpro CRISPR/SaCas9 vector system; Takara). Tumor growth of wild type (WT) and HMGB1-knockout clone D8D3 of B16F10 cells after s.c. transplantation to B6 mice is shown. Each group included 7 mice. The error bars represent the standard error of the mean. (Supplementary materials) [file 12032_2022_1659_MOESM1_ESM.tif]
